# Carbohydrate Microarrays Identify Blood Group Precursor Cryptic Epitopes as Potential Immunological Targets of Breast Cancer

**DOI:** 10.1155/2015/510810

**Published:** 2015-10-11

**Authors:** Denong Wang, Jin Tang, Shaoyi Liu, Jiaoti Huang

**Affiliations:** ^1^Tumor Glycomics Laboratory, SRI International Biosciences Division, 333 Ravenswood Avenue, Menlo Park, CA 94025, USA; ^2^Department of Pharmacology, Weill Medical College of Cornell University, 1300 York Avenue, New York, NY 10065, USA; ^3^Department of Pathology, David Geffen School of Medicine, University of California in Los Angeles, 570 Westwood Plaza, Los Angeles, CA 90095, USA

## Abstract

Using carbohydrate microarrays, we explored potential natural ligands of antitumor monoclonal antibody HAE3. This antibody was raised against a murine mammary tumor antigen but was found to cross-react with a number of human epithelial tumors in tissues. Our carbohydrate microarray analysis reveals that HAE3 is specific for an *O*-glycan cryptic epitope that is normally hidden in the cores of blood group substances. Using HAE3 to screen tumor cell surface markers by flow cytometry, we found that the HAE3 glycoepitope, gp^HAE3^, was highly expressed by a number of human breast cancer cell lines, including some triple-negative cancers that lack the estrogen, progesterone, and Her2/neu receptors. Taken together, we demonstrate that HAE3 recognizes a conserved cryptic glycoepitope of blood group precursors, which is nevertheless selectively expressed and surface-exposed in certain breast tumor cells. The potential of this class of *O*-glycan cryptic antigens in breast cancer subtyping and targeted immunotherapy warrants further investigation.

## 1. Introduction

Recognition of abnormal glycosylation in almost any cancer type has raised great interest in the exploration of the tumor glycome for biomarker discovery [1–3]. In this study, we explored potential glycan markers that are overexpressed on the surfaces of breast cancers. A key immunological probe of this investigation is an antitumor monoclonal antibody (mAb), HAE3. This mAb was raised against epiglycanin, the major sialomucin glycoprotein (~500 kDa) of murine mammary adenocarcinoma TA3 cells [4]. It was initially called AE3 but was later renamed HAE3 [5] to avoid confusion with a commonly used anti-cytokeratin antibody in cancer research [6, 7]. Interestingly, HAE3 was found to strongly cross-react with a number of human epithelial tumors in tissues, including lung, prostate, bladder, esophagus, and ovarian cancers [5, 8–10].

This cross-species tumor binding profile suggests the possibility that HAE3 may recognize a conserved tumor glycan marker that is coexpressed by both mouse- and human-derived epithelial cancers. HAE3 was initially suggested to resemble lectin peanut agglutinin (PNA), which recognizes the T disaccharide (Gal*β*1,3GalNAc*α*1-) linked to Ser/Thr [11]. However, the antibody differed from PNA in that the concentration of the blood group T disaccharide required for inhibition of binding to epiglycanin was 10^4^ times greater than for PNA. Moreover, a T-specific mAb HH8 was found to be negative with epiglycanin in ELISA microtiter plates [12]. HH8 is specific for the T-terminal disaccharide moiety expressed by asialoglycophorin A [13]. Consistent with these observations, Palma et al. reported recently that the neoglycolipid conjugates that display a single T disaccharide epitope were negative with HAE3 [14]. Of note, mAb HAE3 was cited as AE3 in the report.

Given that HAE3 has been analyzed using a large collection of synthetic neoglycoconjugates (*n* = 492) [14], this study focused on identification of the potential natural ligands of HAE3. In essence, we produced a comprehensive carbohydrate microarray using a large collection of purified natural carbohydrate antigens for screening. These include A, B, O, Lewis^a/b^, I and i, and the blood group precursors of various biological origins. As summarized below, we have revealed that HAE3 is specific for a blood group precursor cryptic epitope that is normally hidden in the cores or internal chains of blood group substances but becomes differentially expressed in human breast cancer cells.

## 2. Material and Methods

### 2.1. Antigens and Antibodies

A preparation of human carcinoma-associated antigen (HCA) (1^#^) was kindly provided by Dr. Zeqi Zhou of Egenix (Millbrook, NY). The murine hybridoma IgM antibody, HAE3, was produced by mouse immunization (C57BL/J) with asialoepiglycanin (85^#^) [4, 5]. A preparation of purified HAE3 protein was purchased from RA Biosources, Inc. (Belmont, CA). Carbohydrate antigens applied in this study are listed in Supplementary Table 1 in Supplementary Material available online at http://dx.doi.org/10.1155/2015/510810.

### 2.2. Cell Lines

Cancer cell lines used include breast-derived (T-47D, SK-BR-3, MCF-7, BT-549, MD-AMB-231, and MD-AMB-468), lung-derived (A549), or prostate-derived (PC3) epithelial tumor cell lines and a skin-derived melanoma SKMEL-28. All tumor cell lines were acquired from ATCC.

### 2.3. Carbohydrate Microarrays

**Figure 1:**
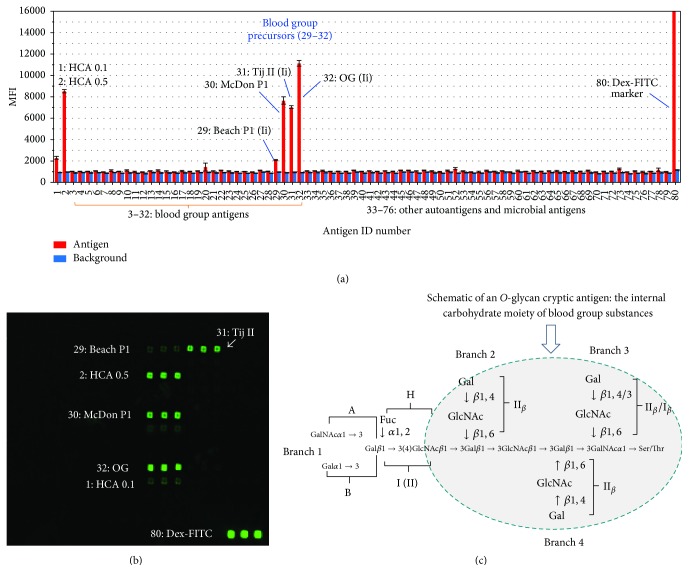
Carbohydrate microarray analysis of antiepiglycanin mAb HAE3. Seventy-six glycoproteins, glycoconjugates, and polysaccharides were spotted in triplicates in 1 to 2 dilutions to yield the customized microarrays for antibody screening. (a) Microarray detections were shown as the mean fluorescent intensities (MFIs) of each microspot with antigen-binding signal in red and background reading in blue. Each error bar is constructed using one standard deviation from the mean of triplicate detections. The labeled antigens include HCA (ID# 1 and ID# 2), a number of blood group precursors (29^#^–32^#^), and a microarray spotting marker (80^#^). (b) Images of a microarray stained with HAE3 (5 *μ*g/mL). (c) Schematic of a blood group substance structure with the conserved* O*-glycan core highlighted.

Microarray assays were performed as described [15]. In brief, a microarray robot (PIXSYS 5500C, Cartesian Technologies, Irvine, CA) was used to spot antigen preparations onto glass slides precoated with nitrocellulose polymer (FAST Slides; Schleicher & Schuell, Keene, NH). The printed microarrays were incubated at room temperature with HAE3 (IgM) antibody at 5 *μ*g/mL in 1% (wt/vol) BSA in PBS containing 0.05% (wt/vol) NaN_3_ and 0.05% (vol/vol) Tween-20. An R-phycoerythrin- (R-PE-) conjugated affinity-purified F(ab′) fragment of goat anti-mouse IgM secondary antibody preparation (Rockland Immunochemicals, Inc., PA) was applied at 2.0 *μ*g/mL to reveal HAE3-specific staining signal. Fluorescence intensity values for each array spot and its background were calculated using ScanArray Express software. SAS Institute's JMP-Genomics software package (http://www.jmp.com/; Cary, NC) was used for microarray data standardization and statistical analysis. Results of the microarray assay are shown as the means of fluorescent intensities (MFIs) of triplicate detections of given antigen preparations (Figure 1(a) and Table 1).

### 2.4. Fluorescence-Activated Cell Sorting (FACS) Analysis

HAE3 (IgM, 5.0 *μ*g/mL) and an isotype control mAb 9.14.7 (IgM, anti-*α*(1,6)dextran, 5.0 ug/mL) [16] were applied to stain tumor cell lines. The R-PE-conjugated goat anti-mouse IgM antibody preparation described above was applied in the second staining step to reveal the antigen-captured IgM. FACS data were collected with LSR-II (BD Bioscience, San Jose, CA) and analyzed with FlowJo (TreeStar Inc., Ashland, OR).

### 2.5. Enzyme-Linked Immunosorbent Assay (ELISA) and ELISA Inhibition Assays

Carbohydrate-specific ELISA and ELISA inhibition assays were performed as described [15, 17]. In brief, glycoprotein antigen preparations were diluted in 0.1 M sodium bicarbonate buffer solution, pH 9.6, for coating on ELISA microplates (NUNC, MaxiSorp, Thermo Scientific, Rochester, NY) followed by blocking using 1% BSA and 1X Phosphate Buffered Saline Tween-20 (PBST). MAb HAE3 (IgM) (2.5 *μ*g/mL) and biotinylated PNA (2.0 *μ*g/mL) were diluted in 1% BSA, PBST for the ELISA binding assay. The bound HAE3 and PNA were revealed by an alkaline phosphatase- (AP-) conjugated goat anti-mouse IgM and an AP-streptavidin conjugate (Sigma Chemical Co., St. Louis, MO), respectively. ELISA inhibition assays were performed with EPGN (85^#^) (1 *μ*U/mL) coated to display the native gp^HAE3^ epitope and a series of blood group reference antigens as potential inhibitors (25.0 *μ*g/mL/each) to compete with the HAE3 (1.0 *μ*g/mL) binding of EPGN. Percent inhibition was calculated as follows: % inhibition = ((standard A − blank A) − (A with inhibitor − A))/(standard A − blank A).

### 2.6. Bio-Gel Chromatography

Bio-Gel P-10 filtration was performed following the manufacture's manual (Bio-Rad Laboratories, Hercules, CA) with minor modifications. In brief, Tij II (20% from 2nd 10%) substance was fractioned in a precalibrated Bio-Gel P-10 column. The sizes of the Tij II substance were measured based on the neutral sugar elution profile with reference to the calibrated saccharide molecular weight standards as shown in Figure 4.

## 3. Results

### 3.1. Carbohydrate Microarray Identifies Blood Group Precursors as the Natural Ligands of HAE3

As shown in Figure 1 and Table 1, an HCA preparation (ID^#^ 1 and 2) was spotted as a positive control for HAE3 activity. This HAE3^+^ glycoprotein preparation was affinity-purified from cultural supernatant of the lung cancer cell line A549 using an HAE3-agarose column (Egenix, Millbrook, NY). Given that the simple* O*-glycan core T disaccharide (Gal*β*1,3GalNAc-) and its peptide conjugates were found to weakly but significantly inhibit HAE3 binding to epiglycanin, we characterized a panel of blood group substances that carry* O*-glycan cores in this microarray screening. A number of blood group precursors (29^#^–32^#^) were plotted from 3^#^ to 32^#^, which was followed by other antigens from 33^#^ to 78^#^ and microarray printing and scanning calibration controls in 79^#^ and 80^#^. Blood group substance reference reagents [18] used include the following: Cyst9 and Cyst14, A active; Beach phenol insoluble, B active; Hog, H active; JS phenol insoluble, H and Le^b^ active; and N-1 20% from the second 10%, Le^a^ active. Key blood group precursor references include OG, Tij II, Beach P1, and McDon P1. These precursor substances were prepared to remove most of the *α*-L-fucosyl end groups that are essential for blood groups A, B, and H or Lewis active side chains but possess the internal domains or core structures of blood group substances. A large panel of other autoantigens and microbial polysaccharides were spotted in the same microarrays to examine potential polyreactivity of this IgM antibody.

Figures 1(a) and 1(b) illustrate a representative result of multiple microarray screening assays. In Figure 1(a), the MFIs of carbohydrate microarray detections of HAE3 binding signal (red column) are plotted with corresponding local background reading (blue column) as an overlay plot. Each data point represents the mean of triplicate detections; these are shown in the Figure 1(b) microarray image with the number of positive antigens labeled. Each error bar is constructed using one standard deviation from the mean. As illustrated, HAE3 is strongly positive with HCA (1^#^ and 2^#^) as expected. Importantly, this antibody selectively binds to four blood group precursor antigens, Beach P1 (29^#^), McDon P1 (30^#^), Tij II (31^#^), and OG (32^#^). By contrast, HAE3 has no detectable cross-reactivity with blood group substances A, B. O, or Lewis antigens, or the large panel of other carbohydrate antigens spotted in the same array.

Figure 1(c) is a schematic of blood group substance structure with the common blood group precursor core structure highlighted. The four-branched structure in the circle represents the internal portion of the carbohydrate moiety of blood group substances, which was proposed based on extensive immunochemical characterization of precursor OG and other P1 fractions of blood group precursors that were isolated from ovarian cancer cyst fluids [19–22]. Selective detection of these blood group precursors from a large panel of blood group substances by HAE3 illustrated that this antibody is specific for a shared cryptic glycoepitope of these precursor substances.

### 3.2. Carbohydrate-Specific ELISA and ELISA Inhibition Assays Validate Binding Specificity of HAE3

**Figure 2:**
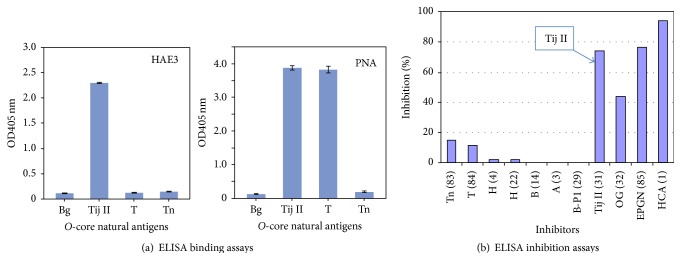
Carbohydrate-specific ELISA and ELISA inhibition assays validate binding specificities of HAE3. (a) An antigen-specific ELISA distinguished HAE3 binding specificity from the T-antigen-specific PNA. ELISA plates were coated with* O*-core antigen, Tij II (31^#^), T (81^#^), and Tn (82^#^) at 10 *μ*g/mL to react with either HAE3 (5.0 *μ*g/mL) or PNA (1.0 *μ*g/mL). (b) ELISA inhibition assays with a series of carbohydrate antigens as competitors (25.0 *μ*g/mL) to inhibit interaction between EPGN (1.0 *μ*U/mL) and anti-HCA (HAE3, 1.0 *μ*g/mL). These antigens include HCA (1^#^), EPGN (85^#^), which is the immunogen of HAE3, and blood group substances Tn (83^#^), T (84^#^), H (4^#^ or 22^#^), A (3^#^), B (14^#^), Beach P1 (29^#^), Tij II (31^#^), and OG (32^#^). Results are shown as percent inhibition in the presence of an inhibitor.

We further examined whether the observed HAE3 binding reactivity can be attributed to cross-reactivity with T/Tn glycoepitopes that are often expressed as components of blood group precursor substances. For this purpose, we tested ELISA binding of HAE3 or T-specific lectin PNA with blood group precursor Tij II (31^#^) and two T/Tn-positive glycoconjugates, asialo-PSM (T) (81^#^) and asialo-OSM (Tn) (82^#^). Unlike PNA, which binds to both Tij II and asialo-PSM (T), HAE3 specifically binds to Tij II without cross-reacting with asialo-PSM (T) or asialo-OSM (Tn) (Figure 2(a)). Thus, HAE3 binding of Tij II is irrelevant to the native T/Tn-glyco-epitopes expressed by these glycoconjugates. ELISA inhibition assays further demonstrated that blood group precursors Tij II (31^#^) and OG (32^#^), but not other blood group antigens, including A (3^#^), B (14^#^), H (22^#^), T (84^#^), or Tn (83^#^), significantly inhibited HAE3 binding to the immunogen asialoepiglycanin (85^#^) (Figure 2(b)).

### 3.3. FACS Analysis Detects Tumor Cell Surface Expression of gp^HAE3^

**Figure 3:**
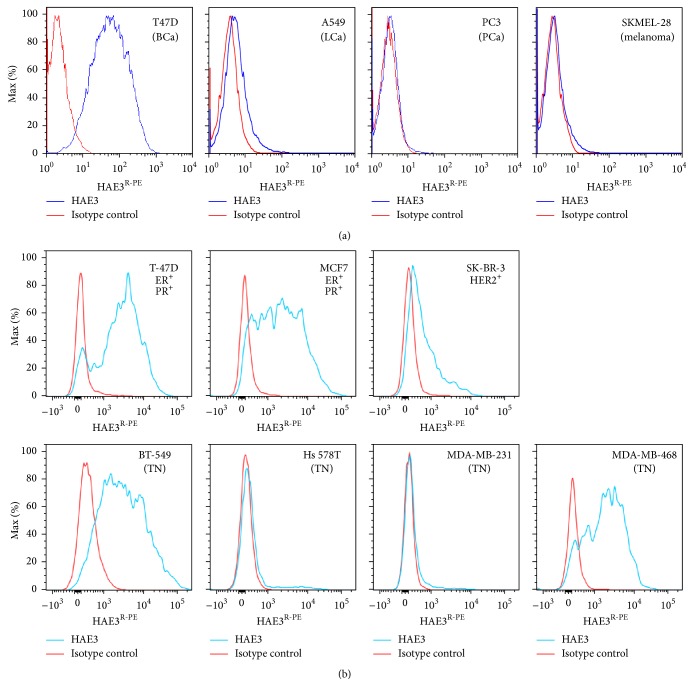
HAE3 cell surface staining detected selective expression of the HAE3-cryptic glycan markers in human cancer cell lines. (a) Four tumor cell lines, T-47D, A549, PC3, and SKMEL-28, were stained with the C1 preparation of HAE3 (IgM) at 1 : 6 dilution or with an isotype control IgM, 9.14.7 (5.0 *μ*g/mL). (b) Seven breast cancer cell lines were stained with purified mAb HAE3 (5.0 *μ*g/mL) or 9.14.7 (5.0 *μ*g/mL). These cell lines are T-47D, MCF-7, SK-BR-3, BT-549, Hs578T, MDA-MB-231, and MDA-MB-468. An R-PE-conjugated goat anti-mouse IgM antibody was applied to quantify the cell surface-captured IgM antibodies.* Blue line*: HAE3 stain;* Red line*: 9.14.7 IgM isotype control.

We examined whether the HAE3^+^ glycoepitopes were expressed as cell surface tumor markers. In the first set of experiments, we screened a panel of four tumor cell lines by cell surface staining in flow cytometry. These include (a) a breast cancer line, T-47D, which was selected owing to the fact that breast cancer patients were found to produce substances in circulation that are highly effective in inhibiting HAE3 binding of epiglycanin [12, 23], (b) a lung cancer line, A549, which is known to produce an HAE3-positive substance in cell culture, (c) a prostate cancer line, PC3, which is found to express a blood group B-related F77 glycoepitope [24, 25], and (d) a melanoma cell line SKMEL-28, which is derived from skin but not epithelial tissue. As shown in Figure 3(a), melanoma SKMEL-28 and prostate cancer PC3 were negative for HAE3. The A549 lung cancer cell line was weakly positive. By contrast, the breast cancer cell line T-47D was strongly positive in HAE3-cell surface staining.

Given these results, we extended the FACS analysis to a panel of seven human breast cancer cell lines, including two ER^+^PR^+^ lines (T-47D and MCF-7), one ER^+^ (SK-BR-3), and four triple-negative cancers (BT-549, Hs 578T, MDA-MB-231, and MDA-MB-468).  Figure 3(b) shows that two ER^+^PR^+^ lines, T-47D and MCF-7, and two triple-negative lines, BT-549 and MDA-MB-468, are gp^HAE3^ strongly positive. SK-BR-3 is gp^HAE3^ intermediately positive. By contrast, the two remaining triple-negative cell lines, Hs578T and MDA-MB-231, were HAE3 negative.

## 4. Discussion

Expression of gp^HAE3^ by human breast cancer has been examined in this study. FACS analyses (Figure 3) revealed that five of the seven breast cancer cell lines are HAE3 positive. These are T-47D, MCF-7, SK-BR-3, BT-549, and MDA-MB-468. BT-549 cells are triple-negative/basal-B mammary carcinoma; MDA-MB-468 cells are known as triple-negative/basal-A mammary carcinoma. The two remaining gp^HAE3-^ triple-negative cell lines, Hs578T and MDA-MB-231, were basal-B mammary carcinoma. It is important to extend this investigation to a cohort of breast cancer patients to examine whether this marker was significantly associated with metastatic breast cancer and breast circulating tumor cells, especially in patients with triple-negative cancer cells that lack specific surface biomarkers.

Our carbohydrate microarray analysis has identified blood group precursor substances as the natural ligands of antibody HAE3. As shown in Figure 1, four well-characterized blood group precursor reference antigens, Beach P1 (29^#^), McDon P1 (30^#^), Tij II (31^#^), and OG (32^#^), were HAE3 positive. OG [19] and Tij II [26] antigens were prepared by pepsin digestion, ethanol precipitation, and solubilization in 90% phenol, followed by fractional ethanol precipitation from phenol. Beach P1 [27] and McDon P1 [28] were obtained as the nondialyzable* O*-cores from partially hydrolyzed blood group antigen, Beach B and McDon A1, respectively. Thus, these blood group precursor substances were prepared to eliminate peripheral glycoepitopes, such as A, B, H, or Lewis antigen-specific epitopes, but preserve their* O*-glycan core structures, leaving a number of cryptic* O*-core epitopes exposed for antibody recognition. Selective detection of these blood group precursors from a large panel of blood group substances by HAE3 demonstrated that this antibody is specific for a shared cryptic glycoepitope of these precursor substances.

**Figure 4:**
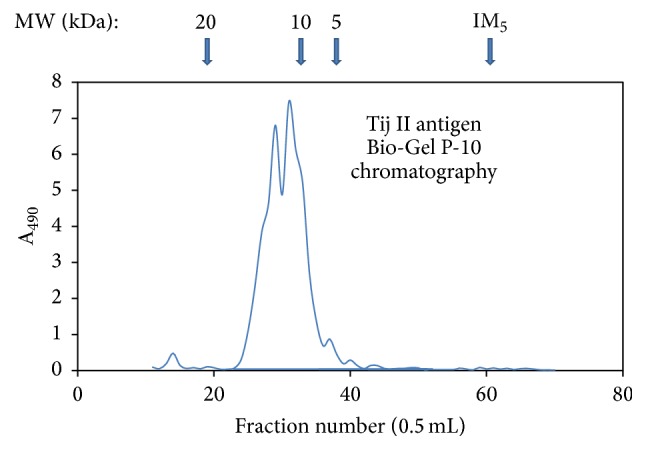
A Bio-Gel P-10 plot of Tij II antigen. Tij II (20% from 2nd 10%) substance was fractioned in a precalibrated Bio-Gel P-10 column at 0.5 mL per fraction. The neutral sugar content in each fraction was determined by phenol-sulfuric acid color reaction and quantitatively measured at OD490 nm. The sizes of the Tij II substance were measured based on the neutral sugar elution profile with reference to the calibrated saccharide molecular weight standards as indicated in the plot. IM_5_ stands for isomaltopentaose.

The native blood group precursor substances are often more complex in carbohydrate structures than the most known model* O*-cores [29]. As determined by Bio-Gel P-10 filtration, the sizes of the Tij II blood group precursor substance were distributed in a range of molecular weights from 7 KDa to 15 KDa, approximately (Figure 4). Since this antigen contains only approximately 2% peptide sequences, its mass is apparently made up predominately of carbohydrates [26, 30, 31]. Figure 1(c) is a postulated blood group precursor core structure, which was proposed based on extensive glycan structural analyses and immunochemical studies of blood group substances [19–22]. The four-branched structure in the circle represents the internal portion of the carbohydrate moiety of blood group substances.

Chemical synthesis of such complex blood group precursors is technically challenging. However, it is not impossible to rationally design and produce HAE3-positive compounds by stabilizing relatively simple* O*-cores via specific structural modifications. For example, a recent microarray screening revealed an unpredicted binding of this antibody to a sulfated glycolipid SM1a, Gal*β*1-3GalNAc*β*1-4(3-O-sulfate)Gal*β*1-4GlcCer [14], which can be viewed as an* O*-core derivative. SM1a naturally occurs in small amounts in normal kidney [32], but such a carbohydrate sequence has not been described in tumor glycome.

Tumor-associated overexpression of blood group-related autoantigens is not limited to breast cancer. Gao et al. recently reported that the natural ligand of a prostate cancer-specific mAb F77 is in fact blood group H, which is built on a 6-linked branch of a poly-N-acetyllactosamine backbone [24, 25]. Overexpression of gp^F77^ in prostate cancers may reflect increased blood group H expression together with upregulated expression of branching enzymes. As illustrated in Figures 1–3, HAE3 differs from F77 in glycan binding specificities and tumor binding profiles. Unlike F77, which is blood group H-specific and stains prostate cancer cell line PC3, HAE3 has neither reactivity with blood group H nor the cell surface targets of PC3.

Both HAE3 and F77 studies call our attention to epithelial tumor expression of blood group substance-related autoantigens. It is noteworthy that blood group substance antigens may also serve as the natural ligands of C-type lectin DC-SIGN, one of the key glycan-binding receptors of the conserved innate immune system [33–35]. Our preliminary data indicates that the HAE3-positive TijII antigen is likely a DC-SIGN ligand (data not shown). Potential of this class of tumor glycoantigens as costimulators of the immune cells in both innate and acquired immune systems for tumor vaccine development and targeted immunotherapy is yet to be explored.

## Supplementary Material

Supplementary Table 1 which summarized antigen ID number, name, source, and key references for each antigen preparation in Supplementary Material.

## Figures and Tables

**Table 1:** Dataset from a carbohydrate microarray analysis of mAb HAE3 (Figure 1).

^*∗*^Antigen spotted		Fluorescent intensities (INT)			Microarray scores (log⁡2-INT)		
(ID#) antigen name	Replicates	Means	StDev	Ag/Bg	Means	StDev	^*∗∗*^ *t*-test (*p* value)
(1)HCA(A549) 1 : 5	**3**	**2306**	**214**	**2.09**	**11.167304**	**0.132563**	**6.66755E − 05**
(2) HCA (A549)	**3**	**8537**	**265**	**7.73**	**13.058996**	**0.044597**	**1.0582E − 07**
(3) Cyst9 (A)	3	1065	5	0.96	10.05708	0.0062627	0.695270476
(4) JS (A)	3	1059	2	0.96	10.048485	0.0027246	0.630836897
(5) HGM-BGS (A + H)	3	1060	12	0.96	10.049785	0.0166116	0.641720796
(6) Hog76 (A)	3	1095	6	0.99	10.096264	0.0072603	0.982754025
(7) Hog (A)	3	996	7	0.90	9.9604633	0.0096233	0.195275968
(8) MSS (A)	3	1139	134	1.03	10.147131	0.1651035	0.701231922
(9) Hog39 B2 (A)	3	1046	10	0.95	10.031084	0.0136387	0.512794031
(10) Cyst14 (A2)	3	1144	116	1.04	10.155053	0.1435773	0.6354829
(11) WG (A2)	3	1021	13	0.92	9.9952239	0.0176786	0.320320938
(12) Cyst11	3	1011	2	0.91	9.9810904	0.002181	0.261637986
(13) Cow21	3	1083	9	0.98	10.080783	0.0122256	0.886644093
(14) Beach (B)	3	1152	83	1.04	10.167059	0.1023248	0.51871479
(15) Cow28 (B)	3	1065	27	0.96	10.056322	0.0369858	0.695618692
(16) Cow43	3	1012	7	0.92	9.9824975	0.0094843	0.267256771
(17) Cow26	3	1093	56	0.99	10.092395	0.0732857	0.985749244
(18) Hog5	3	1057	4	0.96	10.046209	0.0047907	0.614466244
(19) Hog 10%	3	1076	8	0.97	10.071437	0.0104506	0.809324608
(20) Hog6 (H)	3	1462	595	1.32	10.441803	0.5435961	0.384564394
(21) Hog67	3	1067	13	0.97	10.058822	0.0176694	0.709880521
(22) Hog (H)	3	1132	10	1.02	10.144623	0.0123178	0.598292687
(23) Hog30	3	1071	40	0.97	10.064074	0.0539668	0.760857847
(24) Cow21 (I-Ma)	3	1031	7	0.93	10.009339	0.0099403	0.386688505
(25) Cow25	3	1051	24	0.95	10.036836	0.033184	0.555735986
(26) Cow26 (I)	3	1008	13	0.91	9.977683	0.017886	0.250673214
(27) N-1 10% 2X (Le^a^)	3	1139	10	1.03	10.153512	0.0131909	0.537783247
(28) N-1 IO4- (Le^a^)	3	1044	18	0.94	10.0273	0.0250417	0.491711028
(29) Beach P1 (Ii)	**3**	**2119**	**22**	**1.92**	**11.049118**	**0.01476**	**0.000114629**
(30) McDon P1	**3**	**7675**	**604**	**6.95**	**12.903032**	**0.113579**	**4.91829E − 08**
(31) TijII (Ii)	**3**	**7050**	**220**	**6.38**	**12.782943**	**0.044774**	**1.83548E − 07**
(32) OG (Ii)	**3**	**11166**	**433**	**10.10**	**13.446047**	**0.056595**	**2.50433E − 08**
(33) LNT-BSA (Type I)	3	1092	12	0.99	10.092703	0.0152473	0.9870917
(34) Pn XIV (Type II)	3	1108	17	1.00	10.113624	0.02265	0.838256847
(35) ASOR (Tri/m-II)	3	1128	26	1.02	10.138873	0.0330974	0.644502626
(36) AGOR (Tri/m-Gn)	3	1042	5	0.94	10.02559	0.0068188	0.477740236
(37) iAFGP	3	1024	5	0.93	10.000458	0.0070924	0.342706487
(38) Chondroitin-SO4-A	3	1038	13	0.94	10.019053	0.0179351	0.440853108
(39) Chondroitin-SO4-B	3	1128	10	1.02	10.139939	0.0128602	0.631919148
(40) Chondroitin-SO4-C	3	1047	14	0.95	10.031963	0.0189058	0.519441046
(41) Hyaluronic Acid	3	1096	9	0.99	10.098441	0.0113582	0.964347497

(79) Bg	6	1105	188	1.00	10.09423	0.219076	
(80) Dex70K-FITC	3	63225	1067	57.22	15.948071	0.0243011	8.23802*E* − 09

^*∗*^Antigen's initial spotting concentration was 0.5 *μ*g/*μ*L. The positive results are emphasized with bold. Microbial antigens tested (42–78) were negative with HAE3 (Ag/Bg < 1.20; *p* > 0.20, data not shown).

^*∗∗*^ *t*-test: microarray scores, that is, the log⁡2-transformed microarray values from triplicate spots for each antigen, were applied in a *t*-test to examine the differences of significance between each probe and Bg (microarray reading background), which is the mean fluorescent intensity of six spots of Av-Cy3/Cy5 (ID# 79) in the FITC channel.
