# Supplementary material for: Carbohydrate Microarrays Identify Blood Group Precursor Cryptic Epitopes as Potential Immunological Targets of Breast Cancer
Source: J Immunol Res. 2015 Oct 11;2015:510810. doi: 10.1155/2015/510810 (PMC4619957; doi:10.1155/2015/510810)
Supplement: Supplementary file 1 — Supplementary Table 1 which summarized antigen ID number, name, source, and key references for each antigen preparation in Supplementary Material. [file 510810.f1.pdf]

Supplemantary Table 1. Antigen preparations applied in this study

ID#	Antgen preparations (Epitopes)	Description	Source	References
1	HCA (AE3)1:5	Human Carcinoma-associated Antigens (HCA) 1:5 dil.	Egenix (Millbrook, NY)	
2	HCA (AE3)	HCA	Egenix (Millbrook, NY)	Thingstad et al., Eur J Pharm Sci. 6, 121 (1998).
3	Cyst9 (A)	Human ovarian cyst (HOC) substance Cyst 9 Phenol sol., blood group substance A.	From the late Prof. Elvin A. Kabat (Columbia University) (**EAK**)	Baer et al., J. Immunol. 82, 183 (1959); Moreno et al., J. Exp. Med. 134, 439 (1971).
4	JS (H)	HOC substance JS Phenol insol., blood group substance H.	**EAK**	Schiffman et al., Biochemistry. 3,113 (1964).
5	HGM-BGS (A + H)	Hog gastric mucin (HGM), blood group substance A and H.	**EAK**	Feizi et al., J. Exp. Med. 133, 39 (1971).
6	Hog76 (A)	HGM 76, blood group substance A.	**EAK**	
7	Hog 10% (A)	HGM 10%, blood group substance A.	**EAK**	
8	MSS (A)	HOC MSS, blood group substance A.	**EAK**	Moreno et al., J. Exp. Med. 134, 439 (1971);
9	Hog39 B2 (A)	HGM 39 B2, blood group substance A.	**EAK**	Carsten et al., J. Amer. Chem. Soc. 75:3083 (1956).
10	Cyst14 (A2)	HOC Cyst 14, phenol insol dialyzed, blood group substance A2.	**EAK**	Hammarstrom and Kabat, Biochemistry. 8:2696 (1969).
11	WG (A2)	Saliva W. G. phenol insoluble, blood group substance A2.	**EAK**	Moreno et al., J. Exp. Med. 134, 439 (1971).
12	Cyst11	HOC cyst 11 substance.	**EAK**	Kabat, et al, J. Exp. Med. 89, 1 (1949).
13	Cow21	Cow gastric mucin (CGM) substance 21	**EAK**	Beiser & Kabat, J. Immunol. 68, 19 (1952).
14	Beach (B)	HOC Beach, blood group substance B.	**EAK**	Feizi and Kabat, J. Exp. Med. 135, 1247 (1972).
15	Cow28 (B)	CGM substance 28, blood group substance B.	**EAK**	
16	Cow43	CGM substance 43.	**EAK**	Feizi et al., J. Exp. Med. 133, 39 (1971).
17	Cow26	CGM substance 26.	**EAK**	
18	Hog5	HGM substance 5.	**EAK**	
19	Wilson Hog mucin 10%	Wilson HGM substance 10%.	**EAK**	
20	Hog6 (H)	HGM 6, blood group substance H.	**EAK**	
21	Hog67 4% (A)	HGM 67 4%, blood group substance A.	**EAK**	
22	Hog (H)	HGM blood group substance H.	**EAK**	Kabat, et al, J. Exp. Med. 89, 1 (1949);
23	Hog30	HGM substance 30	**EAK**	Beiser & Kabat, J. Immunol. 68, 19 (1952); Allen & Kabat, J. Immunol. 82, 340–357, (1959).
24	Cow21 10% ppt (I-Ma)	CGM substance 21, phenol insoluble,10% precipitations (ppt), blood group substance B+I	**EAK**	
25	Cow25	CGM substance 25, H2O sol.,10% ppt.	**EAK**	
26	Cow26 10% ppt (I)	CGM substance 26, phenol insoluble, 10% ppt, blood group substance I.	**EAK**	Beiser & Kabat, J. Immunol. 68, 19 (1952).
27	N-1 10% 2X (Le^a)	HOC substance N-1 10% 2X, blood group substance Le^a	**EAK**	
28	N-1 IO4- (Le^a)	HOC substance N-1 IO4-,NaOH, blood group substance Le^a	**EAK**	Lloyd et al., Biochemistry 7, 2976 (1968).
29	Beach P1 (Ii, O-Cores)	HOC substance Beach P1 Phenol insol., blood group substance I, i, and O-Cores.	**EAK**	Allen & Kabat, J. Immunol. 82, 358 (1959); Schiffman et al., J. Am. Chem. Soc. 82, 1122 (1959).
30	McDon P1 (O-Cores)	HOC substance McDon P1, Phenol insol., blood group substance precusors.	**EAK**	Sikder et al., Carbohydr Res. 151, 247 (1986).
31	Tij II (Le^a, Ii, and O-Cores)	HOC substance Tij II 20%fr. 2nd 10%, B, Le^a, blood group precusor Ii, T and Tn	**EAK**	Maisonrouge-McAuliffe & Kabat, Arch. Biochem. Biophys. 175, 71 (1976).
32	OG 10% 2X (Ii, O-Cores)	HOC substance OG 10% 2X, blood group precusor I, i, and O-Cores.	**EAK**	Vicari & Kabat, J. Immunol. 102, 821 (1969); Feizi et al., J. Exp. Med. 133, 39 (1971).
33	LNT-BSA (Type I)	Lacto-N-tetraose–BSA	**EAK**	Kabat et al., J. Immunol. 128, 540 (1982).
34	Pn XIV (Type II)	Pneumococcus type XIV capsular polysaccharide	E.R. Squibb and Son, No. 227, Lot 80320	Howe et al., J. Am. Chem. Soc. 80, 6656 (1958).
35	ASOR (Tri/m-II)	Asialo-orosomucoid expressing Tri/m-II glyco-epitopes	**EAK**	Wang & Lu, Physiol Genomics 18(2), 245 (2004).
36	AGOR (Tri/m-Gn)	Agalacto-orosomucoid expressing Tri/m-Gn glyco-epitopes	**EAK**	Wang & Lu, Physiol Genomics 18(2), 245 (2004).
37	iAFGP	Inactive anti-freeze glycoprotein	**EAK**	Feeney et al., J. Biol. Chem. 250, 3344 (1975).
38	Chondroitin sulfate A	Chondroitin sulfate A		
39	Chondroitin sulfate B	Chondroitin sulfate B		
40	Chondroitin sulfate C	Chondroitin sulfate C		Kabat et al., Carbohydr. Res. 130, 289 (1984).
41	Hyaluronic Acid	Hyaluronic acid sodium salt, Sigma-53747	Sigma Chemical Co., St. Louis, MO	http://www.sigmaaldrich.com/catalog

ID#	Antgen preparations	Description	Source	References
42	Helix pomatia	D-Galactans of the snail Helix pomatia	**EAK**	O'Colla, Proc. R. Ir. Acad. Sect. B. Biol. Geol. Chem. Sci. 55B, 165 (1953).
43	Arabino Galactan	Arabino Galactan (Larch CORASH)	**EAK**	Kabat et al., J. Exp. Med. 152, 979 (1980).
44	H.Nemoralis Galactan	D-Galactans of the snail Helix nemoralis	**EAK**	Correa et al., Carbohydr. Res. 3, 445 (1967).
45	Dudman's Rhizobium tritolii TA1	Dudman's Rhizobium tritolii TA1 polysaccharide	**EAK**	Chaudhari et al., Carbohydr. Res. 28, 221 (1973).
46	Pn C	Pneumococcal C polysaccharide (species-specific)	**EAK**	Kabat et al., J. Exp. Med. 152, 979 (1980).
47	Pn SIV	Pneumococcus type IV soluble polysaccharide	**EAK**	Kabat et al., J. Exp. Med. 164, 642 (1986).
48	Pn VIII	Pneumococcus type VIII CP	**EAK**	Brown, R., J. Immunol. 37, 445 (1939).
49	Pn IX	Pneumococcus type IX CP	**EAK**	Kabat et al., J. Exp. Med. 164, 642 (1986).
50	Pn 27	Pneumococcus type 27 CP	**EAK**	Bennett & Bishop, Can. J. Chem. 55, 8 (1977).
51	Bacto-agar 20°C extract	Bacto-agar 20°C extract	**EAK**	Duckworth & Yaphe, Carbohydr. Res. 16, 189 (1971).
52	E. coli. K1	E. coli K1 CP	**EAK**	
53	E. coli. K92	E. coli K92 CP	**EAK**	
54	E. coli. K100	E. coli K100 CP	**EAK**	
55	Phosphomannan	Yeast phosphomannan polysaccharide, NRRL B-2448	Northern Regional research laboratory (NRRL), Peoria, IL	
56	Meningococcus group B	Meningococcus group B CP	**EAK**	
57	Meningococcus group Y	Meningococcus group Y CP	**EAK**	
58	H.influenza A	Haemophilus influenzae type A CP	**EAK**	Kabat et al., J. Exp. Med. 164, 642 (1986).
59	Pn14 ATCC 23-X	Pneumococcal type 14 (US Type 14) CP (ATCC® 23-X™)	Merck, Sharp & Dohme	
60	Pn23 ATCC 25-X	Pneumococcal type 23F (US Type 23) CP (ATCC® 25-X™)	Merck, Sharp & Dohme	
61	Pn23 ATCC 25-X 1:5	Pneumococcal type 23F (US Type 23) CP (ATCC® 25-X™) 1:5 dil.	Merck, Sharp & Dohme	http://www.atcc.org/products
62	Pn23-EAK	Pneumococcal type 23 CP (EAK)	**EAK**	Heidelberger et al., J Immunol.99,794 (1967); Wang et al., Proteomics, 7,180 (2007).
63	Pn23-EAK 1:5	Pneumococcal type 23 CP (EAK) 1:5 dil.	**EAK**	Heidelberger et al., J Immunol.99,794 (1967); Wang et al., Proteomics, 7,180 (2007).
64	S. Weslaco LPS	Shigella Weslaco LPS	**EAK**	Luderitz et al., Bact. Rev. 30, 192 (1966).
65	S. typhi LT2 LPS	Salmonella typhimurium LT2 LPS	**EAK**	Goldman and leive, Eur. J. Biochem. 107, 145 (1980).
66	E.coli K100 CP	E. coli K100 CP	**EAK**	
67	H.influenza A	Haemophilus influenzae type A CP	**EAK**	Kabat et al., J. Exp. Med. 164, 642 (1986).
68	Pn XIV (Gil)	Pneumococcus type XIV CP (Gil)	**EAK**	Allen & Kabat, J. Immunol. 82, 358 (1959).
69	S. dysenteriae I	Shigella dysenteriae Type 1 O-specific polysaccharide	**EAK**	Pozsgay, et al., Proc Natl Acad Sci USA 104:14478 (2007).
70	S. sonnei-1	Shigella sonnei polysaccharide preparation-1	**EAK**	
71	S. sonnei-2	Shigella sonnei polysaccharide preparation-2	**EAK**	Luderitz et al., Bact. Rev. 30, 192 (1966).
72	Pn SII	Pneumococcus type II soluble polysaccharide	E.R. Squibb and Son, No. 227, Lot 80320	Howe et al., J. Am. Chem. Soc. 80, 6656 (1958)
73	Tetanus Toxoid	Tetanus Toxoid from Clostridium tetani	List Biological Labs, Inc., Lot#1919A	http://www.listlabs.com/
74	Isolichenin	α-(1→3)-Glucan	**EAK**	Heidelberger M., J Immunol. 91, 735 (1963).
75	Group C Strep.	Streptococcal group C polysaccharide	**EAK**	Coligan et al., Prog Clin Biol Res. 23, 601 (1978).
76	S. sonnei LPS-3	Shigella sonnei polysaccharide preparation-3	**EAK**	Luderitz et al., Bact. Rev. 30, 192 (1966).
77	S. typhi LPS	Salmonella enterica serotype typhimurium LPS	Sigma-Aldrich	http://www.sigmaaldrich.com/catalog
78	Diphtheria Toxoid	Diphtheria Toxoid, from Corynebacterium diphtheriae	List Biological Labs, Inc., Lot# 1919A	http://www.listlabs.com/
79	Av-Cy3/5	Streptavidin-Cy3 and -Cy5, background fluorescent signal in FITC chanel	Amashan Pharmacia, Piscataway, NJ	http://www.lifetechnologies.com
80	Dex70K-FITC	Positve marker in FITC chanel	Sigma Chemical Co., St. Louis, MO	http://www.sigmaaldrich.com/catalog
81	Asialo-PSM (T)	Prepared from porcine salivary glycoproteins (PSM)	From Prof. Albert Wu, Chang-Gung Mecical Collegue, Taiwan	Tettamanti and Pigman., Arch. Biochem. Biophys., 124:41 (1968).
82	Asialo-OSM (Tn)	Prepared from ovine salivary glycoproteins (OSM)	From Prof. Albert Wu, Chang-Gung Mecical Collegue, Taiwan	Tettamanti and Pigman., Arch. Biochem. Biophys., 124:41 (1968).
83	Tn-antigen-HSA.	Tn-antigen-HSA(7 atom spacer)	V-LABS, INC., NGP2103 (Dec., 2001)	http://www.v-labs.com
84	T-antigen-HSA.	T-antigen-HSA(3 atom spacer)	V-LABS, INC., NGP2208 (Dec., 2001)	http://www.v-labs.com
85	Asialo-epiglycanin (EPGN)	Prepared from EPGN, the major sialomucin glycoprotein (~ 500 kDa) of murine mammary adenocarcinoma TA3 cells	Egenix (Millbrook, NY)	Codington et al., Biochemistry, 11, 2559 (1972)
